# Comparison of psychopathology, purpose in life and moral courage between nursing home and hospital healthcare workers during the COVID-19 pandemic

**DOI:** 10.1038/s41598-024-68983-7

**Published:** 2024-08-07

**Authors:** Iván Echeverria, Lucía Bonet, Ana Benito, Javier López, Isabel Almodóvar-Fernández, Marc Peraire, Gonzalo Haro

**Affiliations:** 1 TXP Research Group, Universidad Cardenal Herrera-CEU, CEU Universities, Castellón de la Plana, Spain; 2 Department of Mental Health, Consorcio Hospitalario Provincial de Castellón, Castellón de la Plana, Spain; 3 Torrente Mental Health Unit, Hospital General Universitario de Valencia, Valencia, Spain; 4 Department of Psychology, School of Medicine, Universidad San Pablo-CEU, CEU Universities, Alcorcón, Spain; 5 Nursing Predepartmental Unit, Universitat Jaume I, Castellón de la Plana, Spain

**Keywords:** COVID-19, Healthcare workers, Moral courage, Nursing home, Purpose in life, Psychopathology, Psychology, Health occupations

## Abstract

The COVID-19 pandemic deeply affected healthcare workers, although the impact may have differed according to different workplace contexts. The aim of this current research was to compare the psychopathology presented by hospital versus nursing home healthcare workers during the COVID-19 pandemic and to analyse the predictive role of purpose in life and moral courage in the appearance of psychopathology. This was an observational, cross-sectional study carried out on a sample of 108 healthcare workers, 54 each from a hospital or nursing homes, who were recruited during the 5 and 6th waves of the COVID-19 pandemic in Spain. Various self-reported scales were used to assess anxiety, depression, acute/post-traumatic stress disorder, drug and alcohol abuse, burnout, purpose in life, and moral courage. Compared to the hospital healthcare workers, nursing home healthcare workers had higher scores and a higher prevalence of anxiety (74.1% vs. 42%), depression (40.7% vs. 14.8%), and post-traumatic stress disorder (55.6% vs. 25.9). In the overall sample, purpose in life was a protective factor against psychopathology (OR = 0.54) and burnout (OR = 0.48); moral courage was a protective factor against depression (OR = 0.47) and acute stress (OR = 0.45); and exposure of family/friends to SARS-CoV-2 was a risk factor for acute stress (OR = 2.24), post-traumatic stress disorder (OR = 1.33), and higher burnout depersonalisation subscale scores (OR = 1.84). In conclusion, the increased presence of psychopathology in nursing home healthcare workers may be influenced by workplace and occupational contexts, personal factors such as exposure of family/friends to SARS-CoV-2, or internal dimensions such as purpose in life and moral courage. This knowledge could be useful for understanding how a future epidemic or pandemic might affect the mental health of healthcare workers in different labour contexts.

## Introduction

On 31 December 2019, the Wuhan Municipal Health Commission (China) reported a cluster of pneumonia cases of unknown origin that were subsequently found to be caused by SARS-CoV-2, the virus responsible for the clinical picture referred to as COVID-19^1^. By the beginning of 2020, the emergency epidemiological crisis resulting from SARS-CoV-2 had overwhelmed Spanish medical resources^2^ and so healthcare workers (HCWs) had to face the situation with a lack of personal protective equipment^3^. Consequently, up to 26% of all people infected with COVID-19 in Spain during the first wave were HCWs, as compared to around 9% in Italy^4^.

### Background

Multiple studies have analysed how the COVID-19 pandemic, as well as work-related factors such as professional category, exposure to an unknown virus, high infection rates, and staff shortages resulting in increased working hours and workload, impacted the mental health of HCWs^5–7^. Indeed, high levels of anxiety, depression, post-traumatic stress disorder (PTSD), and burnout, a dysfunctional response to prolonged work stress characterised by the appearance of emotional exhaustion, depersonalisation, and low personal fulfilment^8^, were reported in Spain^8,9^ and worldwide^10,11^. In this regard, psychopathology has also been linked to the development of burnout^5^ and vice versa^12^. However, fewer studies have explored whether the workplace environment itself (i.e., hospital versus extra-hospital contexts) can affect the appearance of psychopathology and burnout in HCWs.

A relevant example of the extra-hospital context is nursing homes, where HCWs not only had to deal with long work shifts, high workloads, and an elevated risk of contagion^13^, but also suffered from the high number of resident deaths, which amounted to more than 34,000 at the beginning of 2023^14^. This implies that a quarter of all people who died from COVID-19 in Spain were nursing home residents. As a result, nursing home HCWs were at greatest risk of suffering from psychopathology during this time^15^.

In addition to the previous extrinsic factors, various studies have also analysed whether intrinsic factors, such as existential or moral dimensions, are related to the development of psychopathology and burnout in HCWs.

One of these intrinsic dimensions is purpose in life (PIL), understood as the perception each individual has that their life has a purpose and value^16^. PIL is a long-standing concept that was first defined by Viktor Frankl after World War II, and is a key dimension of meaning in life, with both these concepts, in turn, being related to resilience^17^. Although the influence of PIL on psychopathology has been studied previously, high PIL scores were associated with lower levels of anxiety, depression, acute stress, and burnout among HCWs during the COVID-19 pandemic^8,18^.

In this context, another relevant dimension is moral courage (MC), understood as the ability to face danger or social disapproval when one is doing what they consider to be their duty^19^. The role of MC in mental health is complex because it is closely linked to other dimensions such as moral resilience or moral distress and can both protect against and also generate psychopathology. In this sense, the pandemic may have been a key source of moral distress, defined as the dissonance caused by the gap between the moral values of an individual and the behaviour they are ultimately able to perform because of the context^20^. Indeed, high MC was associated with a higher probability of suffering psychopathology during the COVID-19 pandemic^18^.

All these findings seem to indicate that to obtain a more complete view of the psychopathology caused by the pandemic, other influencing variables such as PIL, MC, burnout, and the workplace environment must be considered. However, very few studies have compared the presence of psychopathology and burnout in hospital and nursing home HCWs during the COVID-19 pandemic and none of them considered the roles that PIL and MC may have played in their occurrence.

### Purpose

The objective of this current study was to compare the presence of psychopathology and burnout in hospital and nursing home HCWs during the COVID-19 pandemic and to analyse the possible effects of PIL and MC in this context. We hypothesised that: (1) psychopathology and burnout would be higher in nursing home healthcare workers than in hospital healthcare workers; (2) purpose in life would be a protective factor and moral courage a risk factor for psychopathology and burnout both in nursing home and hospital healthcare workers.

Thus, this study aimed to improve our understanding of the mental health of HCWs in nursing homes during the COVID-19 pandemic, which has been under-researched in most studies. Furthermore, we hoped that analysing PIL and MC in special contexts such as a pandemic would deepen our understanding of the nature and effect of these factors.

## Material and methods

### Study design

Given the research objective and exploratory nature of this work, we conducted a cross-sectional study following the STROBE guidelines for observational studies. Therefore, we limited the inclusion and exclusion criteria so that all participating HCWs had worked during the COVID-19 pandemic and spoke Spanish. G*Power software (v3.1.9.4) was used to estimate the required sample size, considering an expected effect size of *d* = 0.55, an alpha of 5%, and beta of 20% for 2 groups, with an allocation ratio of 1. Hence, we estimated that an overall sample size of 84 or 88 would be required to perform sufficiently powered Student *t* or Mann–Whitney U tests, respectively.

### Recruitment and participants

A convenience and snowball sampling strategy were used to recruit a total of 108 participants, thus ensuring the sample size was sufficient to analyse the variables of interest. The sample comprised 54 HCWs from the Consorcio Hospitalario Provincial de Castellón, the second largest hospital in the city, and another 54 HCWs from several Spanish nursing homes. The cohort included clinical staff (*n* = 75) such as nurses, nursing assistants, and doctors as well as non-clinical staff (*n* = 33), who were mainly administrative workers (*n* = 15), although social workers and cleaning staff, among others, were also included. We considered that both clinical and non-clinical staff had been frontline workers during the COVID-19 pandemic because there had been general uncertainty about which patients were infected, a lack of personal protective equipment, and a high risk of infection among all staff in both Spanish hospitals and nursing homes. The HCW sample from the Consorcio Hospitalario Provincial de Castellón was obtained from a previous study conducted between September and November 2021^8^, while the nursing home HCW data were collected between October 2021 and January 2022, with both periods falling between the 5 and 6^th^ waves of the COVID-19 pandemic in Spain.

### Instruments and data collection

The questionnaires were provided to the HCWs from the Consorcio Hospitalario Provincial de Castellón both in paper and electronic formats between September and November 2021, and because of their multicentric nature, to nursing home HCWs only in an electronic format between October 2021 and January 2022. No differences were expected because the same surveys were sent in both cases. To avoid duplication or fraud with the online surveys, the first and last names of the participants and their work e-mails were collected and the surveyees were assigned an anonymous identification code. This information was encrypted in a separate database which only the principal investigator had access to.

Participants were required to sign their informed consent to participation before commencing the study. All the surveys were self-administered and had been previously validated for Spanish speakers. The questionnaires and methodology were similar to those used in previous studies^8,18,21^.

First, the participants completed a sociodemographic questionnaire that asked about their age, sex, religiosity, marital status, professional category, level of responsibility/role, contract type, time working in their current role, history of physical conditions or mental health disorders, COVID-19 vaccination status, and whether they smoked and the number of cigarettes they smoked.

Second, personal and family/friend exposure to SARS-CoV-2 was assessed using a questionnaire that had been previously employed during the COVID-19 pandemic^18^. PIL was evaluated using the Purpose in Life Test, a 20-item Likert scale test that scores, from 20 to 140, the extent to which each individual considers that their life has a purpose (reliability = 0.89; adequate factorial validity)^16^. This test comprises four dimensions (perception of meaning, experience of meaning, goals and tasks, destiny-freedom dialectic) and has a cut-off point (CP) score of 113, with those exceeding this considered to have a PIL. MC was analysed using the Moral Courage Scale for Physicians (MCSP), a 9-item dichotomous scale that scores, from 0 to 9, the ability of medical personnel or healthcare professionals to face disapproval when doing what they believe is their duty (reliability = 0.74; adequate factorial validity)^22^. The MCSP does not have a CP and higher scores indicate greater MC. The Professional Moral Courage Scale (PMCS), which comprises 12 dichotomous items with a maximum score of 12 (reliability = 0.81; adequate factorial validity)^23^, was also used to analyse MC.

Third, psychopathology and burnout were assessed using various measures described below. Total scores and dichotomous variables were calculated for these scales, and the participants were divided into those who scored above the CP of each scale, thus screening positive for the psychopathology, and those who did not. Anxiety was measured using the Beck Anxiety Inventory (BAI), a 21-item Likert scale with scores ranging from 0 to 63 and a CP of 8 (reliability = 0.90; adequate factorial, discriminant and criterion validity)^24^. Depression was assessed using the Beck Depression Inventory (BDI-II), also a 21-item Likert scale with a CP of 14 (reliability = 0.89; adequate factorial, convergent, discriminant, and criterion validity)^25^. Acute stress disorder was assessed using the Acute/Post-Traumatic Stress Disorder Scale (ETEA-PT), a 15-item Likert scale based on the DSM-5 criteria for these disorders with a CP of 9. An additional ETEA-PT item that asked whether symptoms had lasted more than one month was used to assess PTSD (reliability = 0.81)^18^. Drug abuse was tested employing the Drug Abuse Screening Test-10 (DAST-10), a 10-item dicotomic scale ranging from 0 to 10 with a CP of 1 (reliability = 0.89; proven predictive validity)^26^. Finally, alcohol abuse and problems related to alcohol use were assessed with the Alcohol Use Disorders Identification Test (AUDIT), a 10-item Likert scale ranging from 0 to 40 with a CP of 6 for women and 8 for men (reliability = 0.75; adequate criterion and predictive validity)^27^. The Maslach Burnout Inventory-Human Services Survey (MBI-HSS) was used to evaluate the presence of burnout and its subdimensions. This Likert scale consists of 22 items and comprises three dimensions: personal accomplishment, emotional exhaustion, and depersonalisation (reliability = 0.71, 0.85, and 0.58, respectively)^28^. The score of each subscale is calculated by summing its items and, because of the dimensional complexity of burnout, both the overall score and the scores of one or several subdimensions have been used in the academic literature. Thus, in this current work, the presence of burnout was defined as a high level of either emotional exhaustion (CP ≥ 27) or depersonalisation (CP ≥ 10)^29^.

In addition, a three-item Likert scale was administered to ask participants about their subjective opinion of the change in their mental health since the beginning of the COVID-19 pandemic. Finally, an overall psychopathology score was calculated by summing the absolute scores of each of the psychopathology scales (BAI + BDI + ETEA/TP + DAST-10 + AUDIT).

### Data analysis

First, an exploratory (normality, independence, homoscedasticity, linearity, and non-collinearity) and descriptive study was undertaken. Second, to test hypothesis 1, sociodemographic characteristics, SARS-CoV-2 exposure, PIL, MC, psychopathology, and burnout were compared between the two study groups. Quantitative variables were evaluated using Student *t* and Mann–Whitney U tests (when the assumptions for the application of parametric tests were or were not met, respectively). Categorical variables were compared using Pearson chi-squared test. Third, to test hypothesis 2, generalized linear models and logistic regressions were created for the dependent variables, introducing personal or family/friend exposure to SARS-CoV-2, PIL, and PMCS as predictors. MCSP was excluded from the regression analyses because of collinearity problems with PMCS. Finally, the data were modeled using the PROCESS plugin (v3.4) for SPSS^30^ to study the relationships between the most prominent variables in the regression models. Missing data were eliminated pairwise in each test or analysis.

### Ethical considerations

The ethical principles set out in the Declaration of Helsinki and by the Council of Europe Convention were followed and the informed consent of all participants was obtained. Moreover, data confidentiality was guaranteed according to the General Data Protection Regulation (GDPR; 2018). This study was authorised by the Institutional Review Board (ref. A-15/04/20) and the Clinical Research Ethics Committee (ref. CEI20/068).

## Results

### Participant sociodemographic characteristics

Regarding the sociodemographic characteristics of the participants, the median (Me) sample age was 41 years and 85.2% (*n* = 92) of the participants were women. Significantly more nursing home HCWs (81.5%; *n* = 44) than hospital HCWs (52.8%; *n* = 28) self-identified as religious (χ^2^ = 9.97, *p* = 0.002). In terms of professional category, there were significantly more nurses (38.9%; *n* = 21) in the hospital sample than in the nursing home cohort (13%; *n* = 7), while there were more nursing assistants (48.1%; *n* = 26) in the nursing home group compared to the hospital group (13%; *n* = 7) (χ^2^ = 34.39, *p* < 0.001). Doctors were only present in the sample from the hospital (25.9%; *n* = 14). Significantly more nursing home HCWs included in the cohort (22.2%; *n* = 12) occupied a position of responsibility compared to the hospital HCWs (7.4%; *n* = 4) (χ^2^ = 4.69, *p* = 0.03). Nursing homes stood out because significantly more HCWs (55.6%; *n* = 30) had worked in their current job for 1 to 10 years when compared to the hospital HCWs (23.1%; *n* = 6) (χ^2^ = 9.51, *p* = 0.02). Finally, there were significantly more smokers in the nursing home sample (40.7%; *n* = 22) than in the hospital group (16.7%; *n* = 9) (χ^2^ = 7.64, *p* = 0.006) (Table 1).

**Table 1:** Sociodemographic characteristics of the study participants.

	Total *n* = 108 *n* (%)/Me (IQR)	Hospital *n* = 54 *n* (%)/Me (IQR)	Nursing home *n* = 54 *n* (%)/Me (IQR)	χ^2^/Mann–Whitney U (*p*) CTR
Age	41 (18)	42 (18)	40.5 (19)	1,348.0 (0.85)
Sex (female)	92 (85.2)	46 (85.2)	46 (85.2)	< 0.001 (1)
Religiosity (yes)	72 (67.3)	28 (52.8)	44 (81.5)	9.976 (0.002)** ** − 3.2/3.2**
Marital status				0.442 (0.93)
Single	43 (39.8)	22 (40.7)	21 (38.9)	
Married	53 (49.1)	27 (50)	26 (48.1)	
Divorced	10 (9.3)	4 (7.4)	6 (11.1)	
Widowed	2 (1.9)	1 (1.9)	1 (1.9)	
Professional category				34.394 (< 0.001)***
Nursing assistant	33 (30.6)	7 (13)	26 (48.1)	** − 4/4**
Nurse	28 (25.9)	21 (38.9)	7 (13)	**3.1/− 3.1**
Doctor	14 (13)	14 (25.9)	0 (0)	**4/− 4**
Non-clinical staff	33 (30.6)	12 (22.2)	21 (38.9)	− 1.9/1.9
Role of responsibility (yes)	16 (14.8)	4 (7.4)	12 (22.2)	4.696 (0.03)* ** − 2.2/2.2**
Contract type				7.399 (0.06)
Training	3 (3.8)	3 (11.5)	0 (0)	
Temporary	17 (21.3)	6 (23.1)	11 (20.4)	
Interim/substitute	15 (18.8)	3 (11.5)	12 (22.2)	
Permanent	45 (56.3)	14 (53.8)	31 (57.4)	
Time working in the role				9.511 (0.02)*
< 3 months	4 (5)	3 (11.5)	1 (1.9)	− 1.9/1.9
3 months–1 year	17 (21.3)	8 (30.8)	9 (16.7)	1.4/− 1.4
1 year–10 years	36 (45)	6 (23.1)	30 (55.6)	** − 2.7/2.7**
> 10 years	23 (28.8)	9 (34.6)	14 (25.9)	0.8/− 0.8
Physical condition (yes)	7 (6.5)	2 (3.8)	5 (9.3)	1.316 (0.25)
Mental health disorder (yes)	11 (10.2)	7 (13)	4 (7.4)	0.911 (0.34)
COVID-19 vaccine (yes)	107 (99.1)	54 (100)	53 (98.1)	1.009 (0.31)
Smoker (yes)	31 (28.7)	9 (16.7)	22 (40.7)	7.646 (0.006)**
Number of cigarettes	10 (8)	7 (9)	10 (7)	129,000 (0.20)

n: number of participants; %: percentage; Me: median; IQR: interquartile range; CTR: corrected typified residuals. *p < 0.05; **p < 0.01; ***p < 0.001.

Significant values are in bold.

### Personal and family/friend exposure to SARS-CoV-2, purpose in life, and moral courage

Table 2 shows that there was significantly greater personal and family/friend exposure to SARS-CoV-2 in the nursing home group (Me = 4; interquartile range [IQR] = 3) than in the hospital group (Me = 2; IQR = 3.25) (Mann–Whitney U = 1,831.5, *p* = 0.02). Likewise, the MCSP scale score was significantly higher in the nursing home group (Me = 9; IQR = 1) than in the hospital group (Me = 8; IQR = 2) (Mann–Whitney U = 1,667.5, *p* = 0.009). Surprisingly, there were no differences in PIL between the two groups.

**Table 2:** Personal and family/friend exposure to SARS-CoV-2, purpose in life, and moral courage among nursing home and hospital healthcare workers.

	Total *n* = 108 *n* (%)/Me (IQR)	Hospital *n* = 54 *n* (%)/Me (IQR)	Nursing home *n* = 54 *n* (%)/Me (IQR)	χ^2^/Mann–Whitney U (*p*) CTR
Personal and family/friend exposure	3 (4)	2 (3.25)	4 (3)	1831.5 (0.02)*
PIL (score)	108.6 (17.5)	107.01 (17.4)	110.2 (17.7)	− 0.945 (0.34)
PIL (yes)	48 (45.3)	22 (42.3)	26 (48.1)	0.365 (0.54)
MCSP	8 (2)	8 (2)	9 (1)	1667.5 (0.009)**
PMCS	11 (2)	11 (1.2)	11 (3)	1256.5 (0.78)

n: number of participants; %: percentage; Me: median; IQR: interquartile range; CTR: corrected typified residuals; PIL: Purpose In Life; MCSP: Moral Courage Scale for Physicians, PMCS: Professional Moral Courage Scale. *p < 0.05; **p < 0.01.

Significant values are in bold.

### Psychopathological and burnout variables

Regarding the psychopathology results (Table 3), compared to hospital HCWs, more nursing home HCWs presented anxiety (74.1%; *n* = 40 vs. 42%; *n* = 21) (χ^2^ = 11.01, *p* =  < 0.001), depression (40.7%; *n* = 22 vs. 14.8%; *n* = 8) (χ^2^ = 9.04, *p* = 0.003), acute stress (70.4%; *n* = 38 vs. 25.9%; *n* = 14) (χ^2^ = 21.36, *p* < 0.001), PTSD (55.6%; n = 25 vs. 25.9%; *n* = 14) (χ^2^ = 9.02, *p* = 0.003), and at least one mental health disorder (85.2%; *n* = 46 vs. 56.3%; *n* = 27) (χ^2^ = 10.45, *p* = 0.001).

**Table 3:** Psychopathology and burnout among nursing home and hospital healthcare workers.

	Total *n* = 108 *n* (%)/Me (IQR)	Hospital *n* = 54 *n* (%)/Me (IQR)	Nursing home *n* = 54 *n* (%)/Me (IQR)	χ^2^/t/ Mann–Whitney U (*p*) CTR
BAI	11 (13.7)	6 (10.2)	14.5 (15.5)	1911.5 (< 0.001)***
Anxiety (yes)	61 (58.7)	21 (42)	40 (74.1)	11.013 (0.001)**
BDI-II	7 (11.7)	4 (10)	11.5 (11.2)	2073.5 (< 0.001)***
Depression (yes)	30 (27.8)	8 (14.8)	22 (40.7)	9.046 (0.003)**
ETEA-PT	10 (10.5)	4 (7)	14 (10.2)	2443.0 (< 0.001)***
Acute stress (yes)	52 (48.1)	14 (25.9)	38 (70.4)	21.363 (< 0.001) ***
PTSD (yes)	39 (39.4)	14 (25.9)	25 (55.6)	9.026 (0.003)**
DAST-10	0 (0)	0 (0)	0 (0)	1439.0 (0.25)
Drug (yes)	9 (8.7)	2 (4)	7 (13)	2.63 (0.10)
AUDIT	2 (3)	2.5 (3)	1.5 (2.2)	1226.5 (0.25)
Alcohol (yes)	13 (12.3)	4 (7.7)	9 (16.7)	1.983 (0.15)
Psychopathology (score)	32 (32.5)	20 (26)	43 (33.5)	1892.5 (< 0.001)***
At least one mental disorder (yes)	73 (71.6)	27 (56.3)	46 (85.2)	10.456 (0.001)**
Self-perceived mental health change				11.863 (0.003)**
Improvement	13 (12.1)	11 (20.1)	2 (3.7)	**2.7/ − 2.7**
Decline	54 (50.1)	19 (35.8)	35 (64.8)	** − 3/3**
No change	40 (37.4)	23 (43.4)	17 (31.5)	1.3/-1.3
MBI-HSS (score)	− 20.5 (42.7)	− 23.5 (43.5)	− 18 (43)	1285.5 (0.67)
Burnout (yes)	45 (43.3)	21 (42)	24 (44.4)	0.063 (0.80)
MBI-HSS emotional exhaustion (score)	14 (21)	13 (19)	16 (25)	1504.0 (0.31)
Emotional exhaustion (yes)	26 (25)	11 (22)	15 (27.8)	0.462 (0.49)
MBI-HSS depersonalisation (score)	6 (10)	6 (9)	5.5 (11)	1301.5 (0.75)
Depersonalisation (yes)	37 (35.6)	18 (36)	19 (35.2)	0.008 (0.93)
MBI-HSS personal accomplishment (score)	41.5 (11)	41 (11)	42 (13)	1377.5 (0.85)
Personal accomplishment (yes)	79 (76)	38 (76)	41 (75.9)	< 0.001 (0.99)

n: number of participants; %: percentage; Me: median; IQR: interquartile range; CTR: corrected typified residuals; BAI: Beck Anxiety Inventory; BDI-II: Beck Depression Inventory; ETEA-PT: Acute/Post-traumatic Stress Disorder Scale; PTSD: Post-Traumatic Stress Disorder; DAST-10: Drug Abuse Screening Test-10; AUDIT: Alcohol Use Disorders Identification Test; MBI-HSS: Maslach Burnout Inventory-Human Services Survey. **p < 0.01; ***p < 0.001.

Significant values are in bold.

Likewise, compared to hospital HCWs, nursing home HCWs also had higher scores on the BAI (Me = 14.5; IQR = 15.5 vs. Me = 6; IQR = 10.2) (Mann–Whitney U = 1,911.5, *p* < 0.001), BDI-II (Me = 11.5; IQR = 11.2 vs. Me = 4; IQR = 10) (Mann–Whitney U = 2,073.5, *p* < 0.001), and ETEA-PT scales (Me = 14; IQR = 10.2 vs. Me = 4; IQR = 7) (Mann–Whitney U = 2,443.0, *p* < 0.001), as well as for overall psychopathology score (Me = 20; IQR = 26 vs. Me = 43; IQR = 33.5) (Mann–Whitney U = 1,892.5, *p* < 0.001).

Finally, from the start of the COVID-19 pandemic, compared to hospital HCWs, more nursing home HCWs had perceived a worsening in their mental health (64.8%; *n* = 35 vs. 35.8%, *n* = 19), while more hospital HCWs than nursing home HCWs had perceived an improvement in their mental health status (20.1%; *n* = 11 vs. 3.7%; *n* = 2) (χ^2^ = 11.863, *p* = 0.003) (Table 3).

### Generalized linear models, logistic regressions, and psychopathology data model

There were no differences in the predictors of psychopathology when nursing home and hospital HCWs were analysed separately, except in the case of personal and family/friend exposure to SARS-CoV-2, which could predict ETEA-TP in nursing home HCWs (β = 1.94; 95% CI [1.01, 3.73]; *p* = 0.04) but not in hospital HCWs. Table 4 shows the generalized linear models and logistic regressions used to predict the appearance of a psychopathology or burnout in the overall study sample.

**Table 4:** Generalized linear models and logistic regressions predicting psychopathology and burnout in nursing home and hospital healthcare workers.

Response	Predictors	OR (95% confidence interval) *p*-value
BAI	PIL	0.80 (0.72, 0.89); *p* < 0.001***
BDI-II	PIL	0.77 (0.70, 0.83); *p* < 0.001***
	MCSP	0.47 (0.23, 0.96); *p* = 0.04*
ETEA-PT	PIL	0.90 (0.85, 0.96); *p* = 0.001***
	MCSP	0.45 (0.23, 0.87); *p* = 0.01*
	Personal and family/friend exposure	2.24 (1.19, 4.21); *p* = 0.01*
PTSD	PIL	0.96 (0.93, 0.98); *p* = 0.006*
	Personal and family/friend exposure	1.33 (1.01, 1.75); *p* = 0.03*
DAST-10	–	–
AUDIT	–	–
Psychopathology	PIL	0.54 (0.44, 0.68); *p* < 0.001***
MBI-HSS	PIL	0.48 (0.37, 0.61); *p* < 0.001***
MBI-HSS exhaustion	PIL	0.66 (0.57, 0.77); *p* < 0.001***
MBI-HSS depersonalisation	PIL	0.88 (0.82, 0.93); *p* < 0.001***
	Personal and family/friend exposure	1.84 (1.06, 3.18); *p* = 0.02*
MBI-HSS personal accomplishment	PIL	1.22 (1.13, 1.32); *p* < 0.001***

OR: odds ratio; PIL: Purpose in life; BAI: Beck Anxiety Inventory; BDI-II: Beck Depression Inventory; ETEA-PT: Acute/Post-traumatic Stress Disorder Scale; PTSD: Post-Traumatic Stress Disorder; DAST-10: Drug Abuse Screening Test-10; AUDIT: Alcohol Use Disorders Identification Test. Personal and family/friend exposure, PMCS, and PIL scores were introduced as predictor variables. *p < 0.05; **p < 0.01; ***p < 0.001.

PIL could predict the scores for the BAI (OR = 0.80; 95% CI [0.72, 0.89]; *p* < 0.001), BDI (OR = 0.77; 95% CI [0.70, 0.83]; *p* < 0.001), and ETEA-PT scales (OR = 0.90; 95% CI [0.85, 0.96]; *p* = 0.001), as well as PTSD (OR = 0.96; 95% CI [0.93, 0.98]; *p* = 0.006) and the overall psychopathology score (OR = 0.54; 95% CI [0.44, 0.68]; *p* < 0.001). In addition, PIL could also predict the overall MBI-HSS score (OR = 0.48; 95% CI [0.37, 0.61]; *p* < 0.001), MBI-HSS emotional exhaustion (OR = 0.66; 95% CI [0.57, 0.77]; *p* < 0.001), MBI-HSS depersonalisation (OR = 0.88; 95% CI [0.82, 0.93]; *p* < 0.001), and MBI-HSS personal accomplishment (OR = 1.22; 95% CI [1.13, 1.32]; *p* < 0.001) burnout subscale scores.

In turn, the MCSP could predict the scores on the BDI-II (OR = 0.47; 95% CI [0.23, 0.96]; *p* = 0.04) and ETEA-TP (OR = 0.45; 95% CI [0.23, 0.87]; *p* = 0.001) scales. Finally, personal and family/friend exposure to SARS-CoV-2 could predict PTSD (OR = 1.33; 95% CI [1.01, 1.75]; *p* = 0.03), ETEA-TP (OR = 2.24; 95% CI [1.19, 4.21]; *p* = 0.01), and MBI-HSS depersonalisation burnout subscale scores (OR = 1.84; 95% CI [1.06, 3.18]; *p* = 0.02).

**Figure 1:**
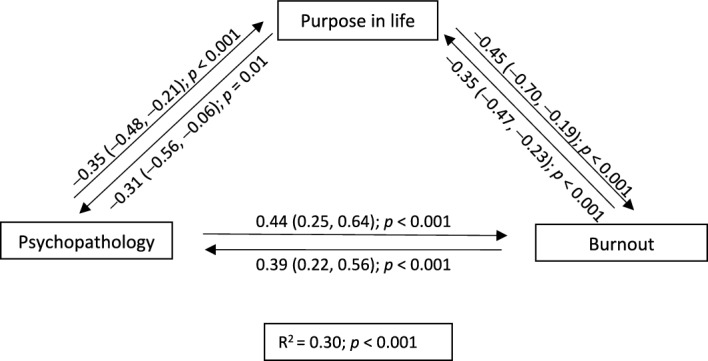
Explanatory model of psychopathology, burnout, and purpose in life.

Given that PIL predicted both psychopathology and burnout in the linear regressions, we modelled these data to learn about the mutual interactions between these variables (Fig. 1). Thus, a reciprocal influence was found between PIL and psychopathology (B =  − 0.31; 95% CI [− 0.56, − 0.06]; *p* = 0.01; B =  − 0.35; 95% CI [− 0.48, − 0.21]; *p* < 0.001), PIL and burnout (B =  − 0.45; 95% CI [− 0.70, − 0.19]; *p* < 0.001; B =  − 0.35; 95% CI [− 0.47, − 0.23]; *p* < 0.001), and psychopathology and burnout (B = 0.44; 95% CI [0.25, 0.64]; *p* < 0.001; B = 0.39; 95% CI [0.22, 0.56]; *p* < 0.001).

## Discussion

To the best of our knowledge, this is the first study to compare the appearance of psychopathology and burnout during the COVID-19 pandemic in nursing home and hospital HCWs, in addition to the predictive role of PIL and MC. Consistent with our first hypothesis, the prevalence of psychopathology was higher in nursing home HCWs than in hospital HCWs, as also previously reported in another Spanish study^15^. Furthermore, nursing home staff were more likely to have reported a deterioration in their mental health since the start of the pandemic. In this sense, professional category may have been one of the main reasons for these differences in psychopathology, because the nursing home cohort had included a higher percentage of nursing assistants. In fact, some studies have suggested that nursing assistants were one of the groups most affected by the COVID-19 pandemic^7,15^. Another reason may have been the high mortality rate (up to 50% of the deaths in the first wave) registered in Spanish nursing homes as a result of COVID-19^15^.

Although our hypothesis of a higher prevalence of burnout among nursing home HCWs was not supported, we demonstrated that burnout and psychopathology were closely related (Fig. 1). In fact, previous studies have shown that burnout can increase the prevalence of psychopathology^31^ and vice versa^12^. However, some extrinsic factors such as personal and family/friend exposure to SARS-CoV-2 may have also played an important role in the appearance of psychopathology and burnout during the COVID-19 pandemic. In fact, this latter risk factor could predict acute stress and PTSD, as well as higher scores on the burnout depersonalisation subscale. Regarding this finding, a previous qualitative study indicated that nursing home HCWs said that one of their main concerns was the transmission of SARS-CoV-2 to their family and friends and that this worry was more stressful to them than contracting the virus themselves^32^. This fear may have led HCWs to feel trepidation when managing residents with COVID-19, which in turn, has been linked to a higher prevalence of PTSD and increased burnout depersonalisation subscale scores^33^, perhaps in response to a dissociative defense mechanism.

In addition to the extrinsic factors mentioned above, intrinsic dimensions such as PIL predicted both the occurrence of psychopathology and burnout, although MC only predicted psychopathology, thereby partially satisfying our second hypothesis. In line with both our second hypothesis and the results of previous studies^8,18^, we observed that high levels of PIL were associated with lower scores for anxiety, depression, acute stress, PTSD, and burnout. These findings could be explained by the fact that PIL is framed within logotherapy and the salutogenic approach to wellbeing^34^. Thus, a high PIL would endow people with greater resilience and coherence in stressful situations, while the opposite situation would be related to a greater likelihood of developing mental health disorders^18,35^. Indeed, a study in nursing home staff during the COVID-19 pandemic showed that low resilience was associated with higher levels of depression^36^.

In contrast to our hypothesis, MC predicted lower depression and acute stress scores in our cohort. Although MC has often been identified as a risk factor for psychopathology because of its association with the concept of ‘moral distress’, it may also be a protective factor against suffering moral distress and, in turn, psychopathology. This ‘double-edged sword’ effect depends on the ability of individuals to act in accordance with their moral expectations^37^, with a failure in being able to do so leading to psychopathology. This phenomenon is also related to the concept of ‘moral resilience’, which refers to the ability to maintain or restore one’s integrity in response to moral adversity. Indeed, moral resilience has been shown to moderate the relationship between exposure to potentially morally distressing events and moral distress and was correlated with lower anxiety and depression in HCWs during the COVID-19 pandemic^38^.

Considering all the above, different authors have proposed several measures to improve crisis management in nursing homes, including the development of personalised action protocols for each site or coordination teams in conjunction with local healthcare services^39^. These measures could reduce the number of deaths among older adults and therefore, reduce the overwhelming work-related situations faced by HCWs that could affect their mental health^40^. The simultaneous development of resources focused on the mental health of HCWs, such as psychological support teams, peer-to-peer programmes, or coping groups, is also recommended. These measures would be useful in the prevention and management of the psychopathology developed during health emergencies^20,41^. In this regard, future management policies should include the systematic and regular assessment of signs of mental disorders in HCWs^9,40^.

Finally, several limitations to this work should be highlighted. First, this was a cross-sectional study, meaning that no inferences regarding causality can be made. Second, since a convenience and snowball sampling strategy were used, the number of people requested to participate in this study and their response rate could not be quantified. Therefore, potential non-response bias or early versus late bias could not be analysed. Furthermore, because of the urgency of the situation caused by the COVID-19 pandemic, measures to mitigate potential common methodological biases could not be implemented. Third, although the hospital sample was drawn exclusively from Castellón, the nursing home sample was recruited from different regions of Spain, leading to a small time lag between the collection of data from the two sample cohorts. Nevertheless, the incidence of COVID-19 in Spain remained broadly the same during both periods and was unlikely to have affected the outcomes. Fourth, although we wanted to address the impact of the pandemic in a naturalistic way by including non-clinical staff, since they may have had less contact with COVID-19, they could be considered non-frontline workers and thus influence the results. Nonetheless, a post hoc analysis to assess the relationship between SARS-CoV-2 exposure and professional categories found no differences between them. Finally, the main study limitation was the differences in sociodemographic characteristics between the two groups, which calls into question the comparability of the groups and role these differences may have played as a significant factor contributing to the results. However, this was an exploratory study with a small sample size compared to the large number of variables studied, thereby leading us to conduct parsimonious analyses. Notwithstanding, it would be interesting to control for these sociodemographic variables in future work in order to discriminate their possible role as confounding variables in the development of psychopathology. Nevertheless, it is still worth highlighting that the variables predicting psychopathology and burnout were almost the same in both groups.

Taken together, these limitations compromise the ability of this work to elucidate the full extent of the influence of PIL and MC on psychopathology or to recommend psychological approaches including these dimensions. Thus, future studies should consider this exploratory work and its limitations when trying to determine the usefulness of PIL and MC as targets of psychological treatments designed to prevent psychopathology.

## Conclusions

The greater presence of psychopathology (anxiety, depression, and post-traumatic stress disorder) in nursing home healthcare workers during the 5 to 6th waves of the COVID-19 pandemic in Spain raises the question of its multifactorial nature and biopsychosocial factors involved in its development.

Regarding extrinsic factors, workplace environment played a central role in the lives of healthcare workers during the pandemic and factors such as professional category may have been of great relevance in the development of mental disorders. Regarding the personal sphere, personal and family/friend exposure to SARS-CoV-2 also played an important role in the appearance of psychopathology. In turn, intrinsic factors including purpose in life or moral courage buffered the effects of nosological elements mentioned above.

Thus, the interaction between the different biological, psychological and social factors specific to each individual (some of which were related to the pandemic) helped configure a latent diathesis that could be activated by a stressor such as a pandemic, ultimately leading to psychopathology. In conclusion, the present research may be useful to start to understand how a future epidemic or pandemic might affect the mental health of healthcare workers in different work contexts and the role of purpose in life and moral courage in the development of psychopathology.

## Data Availability

The data supporting the results of this study are available upon request from the corresponding author. The data are not publicly available because they contain information that could compromise the privacy of research participants.

## Abbreviations

AUDIT: Alcohol Use Disorders Identification Test
BAI: Beck Anxiety Inventory
BDI: Beck Depression Inventory
DAST-10: Drug Abuse Screening Test-10
ETEA-TP: Acute/Post-Traumatic Stress Disorder Scale
HCW: Healthcare workers
MBI-HSS: Maslach Burnout Inventory-Human Services Survey
MC: Moral courage
MCSP: Moral Courage Scale for Physicians
PIL: Purpose in life
PMCS: Professional Moral Courage Scale
